# Primary Hepatic Perivascular Epithelioid Tumor (PEComa) With High Risk of Malignancy: A Case Report

**DOI:** 10.1155/crgm/6695278

**Published:** 2025-10-23

**Authors:** Luis Enrique Quiroga-Hernández, Kharen Alessandra Verjel-Avila, Jerónimo Andrade-Restrepo, Alonso Vera-Torres, Rafael Enrique Andrade-Pérez, Rocío del Pilar López-Panqueva

**Affiliations:** ^1^Department of Pathology and Laboratories, Hospital University Fundación Santa Fe de Bogotá, Los Andes University, Bogotá, Colombia; ^2^School of Medicine, Los Andes University, Bogotá, Colombia; ^3^Department of Surgery, School of Medicine, Hospital University Fundación Santa Fe de Bogotá, Los Andes University, Bogotá, Colombia

**Keywords:** hepatic PEComa, liver, malignancy, perivascular epithelioid tumor, primary

## Abstract

Perivascular Epithelioid Tumors (PEComas) are predominantly present in uterine or gastric tissues. Liver presentations are uncommon and primary hepatic presentations are extremely rare. This is the case of a 31-year-old female patient, with previous diagnosis of hepatic abscess, the patient presented with a one-month history of abdominal pain and bilious emesis. Abdominal MR reported a lesion suggestive of hepatic adenoma. The team performed a partial hepatectomy, and histopathologic and immunohistochemical (IHC) studies reported a PEComa with high risk of confirmed malignant behavior metastatic workup. A complete left laparoscopic hepatectomy was performed due to positive surgical margins. Careful complete histologic and IHC studies are required for diagnosis. IHC reveals coexpression of melanocytic and muscle markers. These studies are usually performed after hepatectomy, the leading management strategy. Long-term follow-up and validated risk stratification scales for hepatic PEComas are needed. This report underscores the critical role of accurate diagnosis and the absence of precise information for management and prognosis of hepatic PEComas.

## 1. Introduction

Perivascular epithelioid cell (PEC) tumors (PEComas) are defined in 2013 by the World Health Organization as a family of “mesenchymal tumors composed of distinctive cells that show a focal association with blood vessel walls and usually express melanocytic and smooth-muscle markers” [1]. This family of tumors is composed by angiomyolipoma, the most common PEComa family tumor in the liver, lymphangioleiomyomatosis (LAM), primary extrapulmonary sugar tumor, clear cell myomelanocytic tumor of the falciform ligament/ligamentum teres [2], abdominopelvic sarcoma of PECs, and tumors with similar features that are simply termed PEComa [3]. Usual anatomical localizations of PEComas are the retroperitoneum, abdominopelvic region, uterus, and gastrointestinal tract [4]. Several body sites with this neoplasm have been reported, including abdominal wall, skin, spinal cord, mediastinum, nasopharyngeal cavity, buccal mucosa, duodenum, bile duct, pancreas, urinary bladder, prostate, penis, breast, uterus, cervix, vulva, ovaries, heart, lung, kidneys, base of skull, ileum, jejunum, colon, rectum, urinary bladder, pelvic wall, ligamentum teres, and falciform ligament [5]. However, hepatic PEComas are very rare, and there are few reports of hepatic malignant PEComa [6]. Herein, we present the experience in diagnosis and treatment of a 31-year-old woman with a primary hepatic PEComa with malignant criteria.

## 2. Materials and Methods

### 2.1. Case Report

A 31-year-old woman was admitted to the emergency department with a one-month history of abdominal pain localized in left hypochondrium that worsen during the last three days, accompanied by bilious emesis. No fever peaks, and other signs of systemic inflammation or changes in depositions and urine were described.

**Figure 1:**
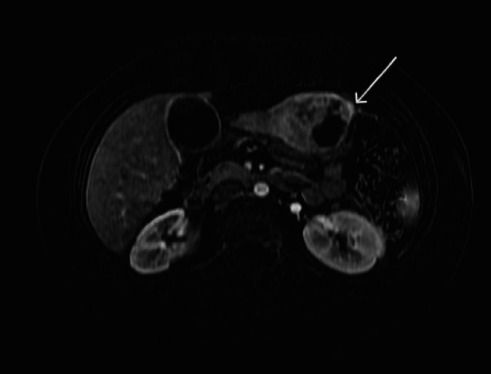
Abdominal MR reporting a focal solid hepatic lesion in Segment III with central cystic components, suggestive of hepatic adenoma.

She was previously diagnosed with a hepatic abscess at another institution but did not respond to medical treatment. Initial abdominal ultrasonography (US) revealed a solid, solitary, heterogeneous mass in the inferior of hepatic Segment III of 48 × 41.3 × 39.8 mm with a hypodense area inside. Additional abdominal magnetic resonance (MR) reported a focal solid hepatic lesion in Segment III of 59 × 57 × 54 mm with central cystic components that is majority restrictive to diffusion, suggestive of hepatic adenoma with no evidence of other abdominal lesions (Figure 1). According to this, she was diagnosed with a hepatic adenoma and continued symptomatic management with acetaminophen. On physical examination, only epigastric and left hypochondrium pain to palpation was evident.

The patient underwent a left laparoscopic Segment III hepatectomy with an uneventful postoperative.

## 3. Results

### 3.1. Pathologic Features

**Figure 2:**
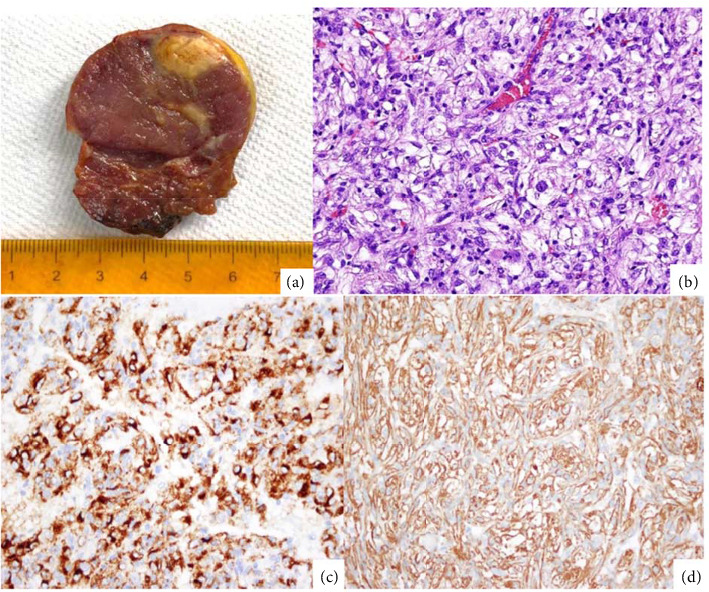
Gross image shows a nonencapsulated, slightly circumscribed mass of 48 × 41.3 × 39.8 mm (a). Histopathology showed epithelioid and spindle cells, moderate nuclear atypia, and cells with clear cytoplasm (40X) (b). The tumor cells are strongly reactive for HMB45 (c) and caldesmon (d).

Following the excision, the specimen had macroscopic evidence of a solid, well circumscribed, homogeneous mass with a larger diameter of 4.5 cm (Figure 2(a)). Frozen biopsy was reported as probable benign tumor with negative margins for malignancy. Furthermore, histopathological study evidenced necrosis, epithelioid and spindle cells, moderate nuclear atypia, and cells with clear cytoplasm. Infiltrative sinusoidal patterns spread to the parenchyma and resection margins. Mitotic figures were > 1 × 50 HPF.

### 3.2. Immunohistochemistry

Immunohistochemical (IHC) studies showed reactivity to CD31–CD34 in endothelium, Melan-A, HMB-45, caldesmon and smooth muscle actin, and there was 20% of KI67 expression (Figures 2(c) and 2(d)). Necrosis, mitosis > 1 × 50 HPF, and infiltrative growth indicated a PEComa of the liver with high risk of malignant behavior. Subsequent thorax computed tomography showed no evidence of additional neoplastic lesions.

Following diagnosis, the patient was scheduled for complete left laparoscopic hepatectomy with satisfactory postoperatory clinical evolution. The patient was discharged with drainage and pain management. Genetic oncology consultation discards PEComa associated to tuberous sclerosis or hereditary predisposition.

## 4. Discussion

Hepatic PEComas are very rare. In a 2015 review, Maebayashi reported 33 primary hepatic PEComa cases [6]. Currently, there are approximately 42 case reports of this pathology, of which, a small group have malignant criteria. Hepatic PEComas occur predominantly in women, with a female-to-male ratio from 2:1 to 5:1, and the age range is 10–86 years [2], with a peak at 30–50 years [5].

The symptomatology is not specific [7]. In general, there is a solitary growing lesion that causes symptoms associated with tumor compression, including abdominal pain, nausea, a dominant palpable mass, and vomiting [8]. The clinical presentation of this patient included abdominal pain and bilious emesis. Because of the nonspecific symptoms, imaging studies must be carried out. However, due to the variable histological appearance, hepatic PEComas lack specific radiological characteristics. On Doppler US, a more vascular flux can aid to diagnosis [9], but in our patient, US revealed a lesion without Doppler flux. Tomography and MR images usually do not allow the accurate differentiation from other hepatic neoplasms or masses [10], but sometimes diffusion MR shows enhancement of the lesion on the arterial phase [11]. In this hepatic lesion, there was restriction to diffusion. The variability on findings may occur because of the variability in the amount of blood vessels.

The pathophysiology of this tumor has not been elucidated yet. Hormones could play an important role, but there is no certainty [12]. Associations between genetic syndromes and hepatic PEComas are scarce. There are some PEComas associated to tuberous sclerosis because of transcription factor E3 (TFE3) gene rearrangements [8]. Additional manifestations suggest that tuberous sclerosis are absent in this patient. Also, the familiar background did not present syndromes with hereditary predisposition to cancer.

Due to the lack of tools for characterization of hepatic PEComas in terms of clinical presentation, imaging studies, and genetic correlation, careful and complete histologic and immunohistochemical studies are required for the final diagnosis.

PEComas are composed of spindle to epithelioid cells, with small, central, round to oval nuclei, with clear to eosinophilic cytoplasm, and sometimes there are changes to clear cells. Rarely, there is melanin pigmentation or fat change [13]. As mentioned above, the amounts of adipose tissue, blood vessels, and muscle cells in PEComas vary from case to case [3]. Immunohistochemical studies show positivity for melanocytic (HMB45, Melan-A, and tyrosinase) and muscular stains (smooth muscle actin, muscle myosin, calponin, and sometimes h-caldesmon) [14]. Principal differential diagnosis can be epithelioid hemangioendothelioma (EHE), paraganglioma, metastatic chromophobe renal cell carcinoma, and adrenocortical carcinoma [15]. In this patient, the key of the PEComa diagnosis was the coexpression of melanocytic and muscle markers.

Behavior of hepatic PEComas is debated due to the rare nature of this pathology. Most case reports show a benign course [16]. However, some cases developed metastasis and recurrence [5]. Dr. Folpe established a three-tier classification system with five high-risk characteristics: tumor size > 5 cm, mitotic rate > 1/50 HPF, infiltrative growth patterns with high nuclear grades and cellularity, vascular invasion, and necrosis. A tumor with two or more features should be considered as malignant, any tumor with one feature has uncertain malignant potential, and benign tumors do not have these characteristics [13, 17]. The accuracy of Folpe's criteria has not been confirmed yet because of the limited number of PEComa cases [18]. Therefore, there is not a standard to classify the malignancy of hepatic PEComas [5]. According to criteria mentioned above, the hepatic PEComa malignancy of this patient could be classified as tumor with high risk of malignancy.

Regarding the prognosis, Bleeker et al. suggest that a high mitotic rate and a size ≥ 5 cm of the primary tumor are associated with recurrence after resection [8]. Other authors have found association between local recurrence or metastasis and parameters including size > 7–8 cm, necrosis, and mitotic index > 1/50 HPF. Some histological findings are indicators of malignancy and poor prognosis for other authors, including infiltrative growth, hypercellularity, pleomorphism, and nuclear atypia [19]. Due to the absence of information about the prognosis, the moderate nuclear atypia and infiltrative growth of this hepatic PEComa have uncertain significance.

Standard treatment is surgical resection of primary tumor and local recurrence or distant metastasis [20]. We performed a laparoscopic Segment III hepatectomy upon the suspicion of a hepatic adenoma. On first intervention, the patient obtained positive margins, whereby a complete left laparoscopic hepatectomy was required.

## 5. Conclusions

We presented a case of primary hepatic PEComa with uncertain malignant potential in a 31-year-old woman. Because of the uncertain prognosis and behavior of this pathology, long-term follow-up will be needed in this patient. Research about pathophysiology, diagnosis, management, and prognosis of hepatic PEComas is needed in order to create valid malignancy criteria and stratification risk scales. Finally, this case report emphasizes the importance of histopathologic and immunohistochemical studies to diagnose hepatic PEComas in the absence of current accurate tools.

## Data Availability

The datasets generated and/or analyzed during the current study are not publicly available due to personal data from the case report but are available from the corresponding author on reasonable request. Correspondence: Rocío del Pilar Lopez Panqueva, MD, Department of Pathology and Laboratory Medicine, Fundación Santa Fe de Bogotá, Carrera 7 #117–15, Bogotá D.C (e-mail: rocio.lopez@fsfb.org.co).
